# Supplementary material for: Cerebellar transcranial direct current stimulation disrupts neuroplasticity of intracortical motor circuits
Source: PLoS One. 2022 Jul 12;17(7):e0271311. doi: 10.1371/journal.pone.0271311 (PMC9275832; doi:10.1371/journal.pone.0271311)
Supplement: Attachment — Submitted filename: Liao_PLOS one_CB-M1_response_document.docx [file pone.0271311.s001.docx]

**Summary**

In response to comments of the two reviewers, we have included some important limitations (Reviewer 1), provided an alternative explanation for elements of our results (Reviewer 1) and clarified our methodological approach (Reviewer 1 & 2). We believe these changes have significantly improved the quality of the manuscript, and thank the reviewers and editor for their time and effort in considering our submission.

**Response to Reviewer 1.**

1. *For the iTMS protocol, can the authors justify why they selected to target the I3-wave (at 4.5 ms) as opposed to (or in addition to) selecting an interval that would predominately target the I2-wave? Since both the I2- and I3-wave get lumped together as “late I-waves”, should we expect a similar finding if one were to target the I2-wave?*

*Response:*

Our decision to target the I3-wave stemmed from existing physiological and functional evidence suggesting that cerebellum may target the circuits responsible for its generation (see paragraph 3 of introduction). In contrast, we are only aware of a single study that has suggested cerebellar interaction with the I2-wave (Ates, Alaydin & Cengiz 2018). Consequently, we felt that focussing on I3 circuits was a more justified approach. While the Ates study suggests that a similar effect on I2 circuits may be possible, the current understanding of these circuits is very limited; indeed, we are unaware of any study that has targeted this interval with iTMS. Consequently, we would prefer to avoid speculating about how these circuits may interact with cerebellum. However, the reviewer makes a good point about the ambiguity with which these two waves are lumped together. We have therefore modified terminology in the revised manuscript (where appropriate) to specifically refer to I3-waves. We have also included a comment about the need for future research to investigate I2-waves more specifically (page 19).

2. *The problem with not conducting this additional control condition is that we don’t really know the reliability and reproducibility of iTMS. For instance, what do the authors believe is the error of margin of using the ISI of 4.5 ms for targeting the I3-wave? For instance, Long et al., 2017 (10.1093/brain/awx102) used an ISI of 4.3 ms for their late I-wave protocol, and the authors of this manuscript previously show that an ISI of 4 ms and 5ms yielded greater facilitation on M1 excitability when compared to 4.5ms. Thus, it seems that another potential interpretation of the results could be that the lack of changes on intracortical excitability and corticospinal excitability might be due to the variability of the “late” iTMS protocol and not influences from the cerebellum.*

*Response:*

For the effect of iTMS on PA responses, our previous work (Opie, Cirillo & Semmler 2018; Opie et al. 2021) suggests facilitation in response to a range of ISIs associated with the late I-wave (including 4, 4.1, 4.5, 4.9 and 5 ms) albeit with a reduced response at 4.5ms, as suggested by the reviewer. This response is supported by the facilitation of PA MEPs following iTMS*_4.5_* in the current study. Consequently, we believe that iTMS targeting the late I-wave is able to reliably modulate PA responses. However, the effects of iTMS*_4.5_* on AP responses have not been as well established, and we therefore cannot exclude the possibility that the insensitivity of AP responses stemmed from variable effects of iTMS*_4.5_* on these circuits. This has been acknowledged in the revised manuscript (page 17).

3. *Why did the authors select cathodal cerebellar tDCS as their cerebellar neuromodulatory technique as opposed to cerebellar theta burst protocol? Cerebellar tDCS does appear to exclusively produces changes in cerebellar excitability and not corticospinal activity (Galea et al., 2009; 10.1523/JNEUROSCI.2184-09.2009), which has been suggested to produce tonic changes at the Purkinje cell level without activating cerebellar-thal-M1 pathways. However, cerebellar theta burst does yield changes in M1 excitability (10.1016/j.clinph.2008.08.008; 10.1038/srep36191), which might reflect this protocol depolarizing cerebellar Purkinje cells and engaging cerebellar-thal-M1 pathways (that produce changes in M1). Thus, one could presume that cerebellar theta burst would have yielded a more disruptive effect on the iTMS protocol.*

*Response:*

We agree that there is greater evidence supporting the influence of cerebellar TBS on M1 excitability, particularly post-stimulation. However, an ability of cerebellar tDCS to influence M1 activity, both during and after stimulation, has also been shown (Ates, Alaydin & Cengiz 2018; Hamada et al. 2014). Within the current study, we reasoned that coincident application of stimulation over cerebellum and M1 would optimise the chance of observing interactions between these areas. Given that iTMS and tDCS both require ~15 minutes of stimulation, whereas TBS involves 40-190s of stimulation, we therefore decided to apply tDCS. We have clarified this reasoning in the revised manuscript (page 3).

4. *While I agree with that different currents are likely recruiting different population of neurons, the previous results by the authors demonstrating changes in AP MEPs following iTMS performed with PA currents (ref 44) does not seem to exactly fit with this notion. One would rather expect that iTMS with PA currents produces greater facilitation of PA MEPs than AP MEPs if distinct populations are being targeted with directional TMS. Thus, if the authors are suggesting that cerebellar tDCS targets AP circuits, would it not be more reasonable to perform iTMS with AP currents?*

*Response:*

Thanks to the reviewer for their comment, we agree that this concept is a problem, and is something we are currently attempting to address in other work. However, this idea is complicated by the higher stimulus intensities required during AP stimulation. This means that it is very unlikely that AP stimulation produces isolated recruitment of AP circuits. This non-specific recruitment is one potential explanation for why we see a potentiation of AP MEPs following PA iTMS (i.e., AP stimulation may be recruiting PA circuits). While the response of AP iTMS to cerebellar modulation would be interesting to investigate, this paradigm has not been characterised previously and will require significant development. It was not feasible to do this within the current study, and we therefore chose to focus on the response to PA iTMS as a preliminary step. Despite this, we have acknowledged this issue in the revised manuscript (page 18).

5. *Why did the authors select only 12 trials for each SICF measure, as opposed to 15 trials for each CBI measure and 20 trials for 1 mV assessment? Is SICF considered more reliable than these other measures to justify the much smaller sample?*

*Response:*

The number of trials included in each condition was based on previous literature (Fernandez et al. 2018; Koch et al. 2008; Opie, Cirillo & Semmler 2018). While we are unaware of formal comparisons of reliability between SICF and CBI, our experience with these measurements would suggest greater reliability of SICF. For example, it is unusual for a participant not to show facilitation with SICF, whereas a lack of inhibition following CBI is far more common.

6. *I think it would be useful for the authors to mention how many individuals could not withstand the cerebellar stimulation at 70% MSO, and if any individuals displayed evidence of antidromic activation. Moreover I suggest the authors to also add this reference for cerebellar stimulation comfort and reliability:* [*https://doi.org/10.1016/j.brs.2019.09.005*](https://protect-au.mimecast.com/s/mzIWCANpk2FrLG1RTOcADH?domain=doi.org)

*Response:*

As suggested, the revised manuscript now lists the number of individuals who could not tolerate CB stimulation at 70%MSO and whether any individuals displayed antidromic activation (page 7).

Thanks to the reviewer for suggesting this reference, it has been included in the revised manuscript.

**Response to Reviewer 2.**

1. *Although the limitation and the conclusion look carefully reported in the Discussion part, the conclusion in the Abstract reads too strong. The conclusion should be weakened not to mislead the readers' interpretation.*

*Response:*

As requested, we have toned down the conclusions included in the abstract.

2. *The reason why the authors selected downregulating cerebellar stimulation (i.e., cathodal tDCS) is unclear. The authors would better carefully explain the rationale why cathodal but not anodal was selected.*

*Response:*

One potential outcome from the current study was to inform the design of interventions that could facilitate targeted manipulation of motor functions by selectively modulating specific intracortical circuits of M1. Consequently, we required an intervention that would theoretically potentiate plasticity in M1. As the inhibitory effects of cerebellum on M1 would be expected to reduce the neuroplastic response, we therefore selected an intervention that would reduce cerebellar excitability (i.e., cathodal tDCS). We have clarified this in the revised manuscript (page 3).

3. *How many trials (MEP samples) were finally excluded from the analysis due to voluntary contraction prior to TMS application? The exact number or percentage should be reported in the manuscript.*

*Response:*

The percentage of excluded MEP trials is now reported in the revised manuscript (page 9).

4. *The information of Fig. 2B and C seems redundant, basically the same as Fig. 2A. The authors should better explain why they needed to perform these additional analyses and prepare these figures. Otherwise, they may better be removed for the sake of simplicity.*

*Response:*

The reviewer makes a good point. We have removed Fig. 2B and included an explanation for the additional analysis (page 12) for Fig. 2C (now relabelled Fig. 2B).

References

Ates, MP, Alaydin, HC & Cengiz, B 2018, 'The effect of the anodal transcranial direct current stimulation over the cerebellum on the motor cortex excitability', *Brain Research Bulletin*, vol. 140, 2018/06/01/, pp. 114-119.

Fernandez, L, Major, BP, Teo, WP, Byrne, LK & Enticott, PG 2018, 'The Impact of Stimulation Intensity and Coil Type on Reliability and Tolerability of Cerebellar Brain Inhibition (CBI) via Dual-Coil TMS', *Cerebellum*, vol. 17, no. 5, Oct, pp. 540-549.

Hamada, M, Galea, JM, Di Lazzaro, V, Mazzone, P, Ziemann, XU & Rothwell, JC 2014, 'Two distinct interneuron circuits in human motor cortex are linked to different subsets of physiological and behavioral plasticity', *Journal of Neuroscience*, vol. 34, no. 38, pp. 12837-12849.

Koch, G, Mori, F, Marconi, B, Codecà, C, Pecchioli, C, Salerno, S, Torriero, S, Lo Gerfo, E, Mir, P, Oliveri, M & Caltagirone, C 2008, 'Changes in intracortical circuits of the human motor cortex following theta burst stimulation of the lateral cerebellum', *Clinical Neurophysiology*, vol. 119, no. 11, Nov, pp. 2559-2569.

Opie, GM, Cirillo, J & Semmler, JG 2018, 'Age-related changes in late I-waves influence motor cortex plasticity induction in older adults', *Journal of Physiology*, vol. 596, no. 13, pp. 2597-2609.

Opie, GM, Sasaki, R, Hand, BJ & Semmler, JG 2021, 'Modulation of Motor Cortex Plasticity by Repetitive Paired-Pulse TMS at Late I-Wave Intervals Is Influenced by Intracortical Excitability', *Brain Sciences*, vol. 11, no. 1, Jan 18.
