# Supplementary material for: Cerebellar transcranial direct current stimulation disrupts neuroplasticity of intracortical motor circuits
Source: PLoS One. 2022 Jul 12;17(7):e0271311. doi: 10.1371/journal.pone.0271311 (PMC9275832; doi:10.1371/journal.pone.0271311)
Supplement: Attachment — Submitted filename: Response to Reviewers_R2.docx [file pone.0271311.s002.docx]

**Response to Reviewer 1**

1. *My final suggestion would be for the authors to include two recent papers in the introduction paragraph #3, which have shown the interaction of cerebellar projections to specific excitatory (DOI: 10.1523/JNEUROSCI.2397-19.2020) and inhibitory (DOI: 10.1016/j.brs.2021.08.022) interneurons.*

Response:

The reviewers *J Neurosci* article has now been referenced in paragraph 3 of the introduction. Regarding the *Brain Stimul* article, while we agree it supports the concept that cerebellum has specific interactions with different intracortical circuits in M1, the implications with respect to early vs late I-waves remain less clear. Given that the point of this paragraph is to describe the evidence supporting differential effects of cerebellum on early and late I-wave circuits, we would therefore prefer to not include reference to this article in this paragraph.

**Response to Reviewer 2**

1. *The rationale for selecting cathodal but not anodal remains unclear. The authors explained in the revised manuscript that “previous work has reported that tDCS to CB modulates concurrent plasticity induction of M1 [15: Hamada et al., 2014]” followed by “therefore, we investigated whether enhancement of M1 plasticity could be achieved by downregulating CB excitability using cathodal tDCS~”. Hamada et al. (2014) demonstrated that concurrent anodal cerebellar tDCS abolishes M1 plasticity, without testing with cathodal one. Hamada et al. (Journal of Physiology, 2012), on which Hamada et al. (2014) rely, also found that both anodal and cathodal cerebellar tDCS block M1 plasticity. These previous findings do not look directly support the authors’ decision to select cathodal but not anodal. A more careful and logical explanation should be achieved.*

Response

The outcomes reported by Hamada and colleagues related to the response to paired-associative stimulation (PAS), and were suggested to be mediated by tDCS (both cathodal and anodal) over cerebellum interrupting afferent input that underpins the spike-timing dependent response to PAS. Consequently, these responses were not thought to be driven by changes to how cerebellum influences motor cortex (M1) directly, but by tDCS over cerebellum influencing afferent input to M1. In contrast, the iTMS paradigm we applied is not dependent on afferent input that might be influenced by cerebellar tDCS, and this was not how we were trying to influence M1 plasticity (we discuss this point on page 15 of the manuscript). Subsequently, we did not expect that cerebellar tDCS within our study would influence M1 plasticity in the same way as was reported by the Hamada studies. Instead, the goal of our intervention was to modulate the direct influence of cerebellum on M1. Specifically, cerebello-thalamo-cortical projections to M1 have an inhibitory effect, and it was therefore possible that reducing this inhibitory tone could produce an environment within M1 that was more amenable to neuroplasticity induction. As previous work has shown that cathodal tDCS over cerebellum is able to achieve this (i.e., CBI is reduced by cerebellar cathodal tDCS; Galea et al., 2009), we decide to apply cathodal stimulation. We have clarified this rationale within the revised manuscript (page 3/4). Furthermore, as we understand why reference to the Hamada papers would have appeared counter intuitive, reference to these papers has been removed from paragraph 2 of the introduction.

2. *Is 1.9% the average of excluded MEPs among participants? Or, is it the percentage of pooled MEPs among all participants and conditions, etc.? Please specify in more detail.*

Response:

This represents the percentage of MEPs excluded across all participants. This has been clarified within the revised manuscript.
